# A Comparison of Multivariate Genome-Wide Association Methods

**DOI:** 10.1371/journal.pone.0095923

**Published:** 2014-04-24

**Authors:** Tessel E. Galesloot, Kristel van Steen, Lambertus A. L. M. Kiemeney, Luc L. Janss, Sita H. Vermeulen

**Affiliations:** 1 Department for Health Evidence, Radboud university medical center, Nijmegen, The Netherlands; 2 Systems and Modeling Unit, Montefiore Institute, University of Liège, Liège, Belgium; 3 Bioinformatics and Modeling, GIGA-R, University of Liège, Liège, Belgium; 4 Department of Urology, Radboud university medical center, Nijmegen, The Netherlands; 5 Department of Molecular Biology and Genetics, Aarhus University, Aarhus, Denmark; 6 Department of Human Genetics, Radboud university medical center, Nijmegen, The Netherlands; Institute of Cytology & Genetics SD RAS, Netherlands

## Abstract

Joint association analysis of multiple traits in a genome-wide association study (GWAS), *i.e*. a multivariate GWAS, offers several advantages over analyzing each trait in a separate GWAS. In this study we directly compared a number of multivariate GWAS methods using simulated data. We focused on six methods that are implemented in the software packages PLINK, SNPTEST, MultiPhen, BIMBAM, PCHAT and TATES, and also compared them to standard univariate GWAS, analysis of the first principal component of the traits, and meta-analysis of univariate results. We simulated data (N = 1000) for three quantitative traits and one bi-allelic quantitative trait locus (QTL), and varied the number of traits associated with the QTL (explained variance 0.1%), minor allele frequency of the QTL, residual correlation between the traits, and the sign of the correlation induced by the QTL relative to the residual correlation. We compared the power of the methods using empirically fixed significance thresholds (α = 0.05). Our results showed that the multivariate methods implemented in PLINK, SNPTEST, MultiPhen and BIMBAM performed best for the majority of the tested scenarios, with a notable increase in power for scenarios with an opposite sign of genetic and residual correlation. All multivariate analyses resulted in a higher power than univariate analyses, even when only one of the traits was associated with the QTL. Hence, use of multivariate GWAS methods can be recommended, even when genetic correlations between traits are weak.

## Introduction

Genome-wide association studies (GWAS) have been very successful in the identification of common genetic variants associated with complex traits. Usually, information on a set of related traits is collected in populations sampled for GWAS. These traits are typically analyzed separately, *i.e*. in a univariate manner, for association to genome-wide DNA markers. This is often followed by an informal comparison of evidence for association at particular loci across the studied traits (*e.g*. [1]). However, a joint analysis of multiple, potentially correlated traits, *i.e*. a multivariate analysis, could be very advantageous for a number of reasons. First, a multivariate analysis has increased power in case of presence of genetic correlation between the different traits; the extra information that is provided by the cross-trait covariance is ignored in univariate analyses [2], [3]. Secondly, most multivariate procedures can perform a single test for association with a set of traits. This reduces the number of performed tests and alleviates the multiple testing burden compared to analyzing all traits separately [2], [4]. Finally, in case of presence of pleiotropy, where a single genetic variant is associated with multiple traits, a multivariate GWAS is more consistent with biology compared to cross-trait comparison of univariate analyses [5].

**Figure 1:**
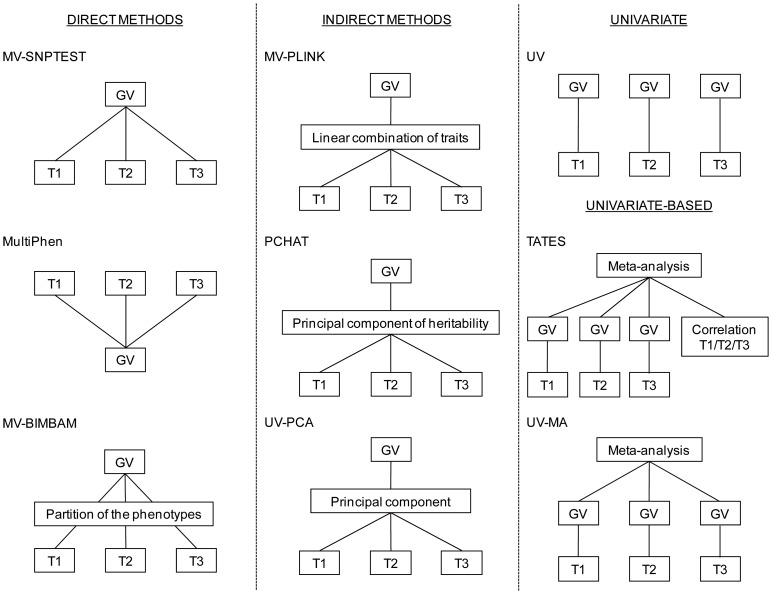
Schematic representation of the included methods. GV indicates genetic variant; MV, multivariate; PCHAT, Principal Component of Heritability Association Test; T1, trait 1; T2, trait 2; T3, trait 3; TATES, Trait-based Association Test that uses Extended Simes procedure; UV-MA, meta-analysis of univariate results; UV-PCA, univariate analysis of first principal component.

A number of methods for simultaneous analysis of multiple traits in population-based GWAS have been published (*e.g*. [4], [6]–[19]). Although a few of the methods have been compared to newly proposed methods [12], [15] and some of the methods have been compared to univariate analysis [4], [7], [12], little is known about their relative performances. Here, we performed the first direct comparison of several multivariate (MV) GWAS methods using simulated data. We included six methods, with a focus on methods already implemented in freely available software: the multivariate test of association MQFAM implemented in the genetic association analysis software PLINK (MV-PLINK) [7], a Bayesian multiple phenotype test implemented in SNPTEST (MV-SNPTEST) [20], the R package MultiPhen (MultiPhen) [12], a Bayesian model comparison and model averaging for multivariate regression in BIMBAM (MV-BIMBAM) [21], [22], the Principal Component of Heritability Association Test (PCHAT) [4], and a Trait-based Association Test that uses Extended Simes procedure (TATES) [15]. These can be classified into direct, indirect and univariate-based methods (Figure 1). MV-SNPTEST, MultiPhen and MV-BIMBAM are direct MV methods, in which the effects of the genetic variant are modeled directly on the traits without changing the general format and nature of the trait data. MV-SNPTEST [20] and MV-BIMBAM [22] are both based on a Bayesian multivariate regression analysis, but MV-BIMBAM additionally partitions the traits into three groups: 1) traits that are unaffected by the genetic variant, 2) traits that are directly affected by the genetic variant, and 3) traits that are indirectly affected by the genetic variant through directly affected traits. MultiPhen identifies the linear combination of traits most associated with each genetic variant by applying a reversed ordinal regression, such that genotype (allele count) is regressed on a collection of traits [12]. MV-PLINK [7], PCHAT [4] and UV-PCA are indirect methods based on a reduction of the trait dimension. In MV-PLINK the association between a set of traits and a genetic variant is assessed using canonical correlation analysis. Specifically, the linear combination of traits that maximizes the covariance between the genetic variant and all traits is extracted. PCHAT is based on extracting the principal component of heritability that is the optimal linear combination of the traits from a heritability point of view [4]. In TATES [15], the observed correlation structure between the traits is taken into account in the meta-analysis approach.

We compared the power of the methods under empirically fixed type I errors to one another and to standard univariate (UV) analysis, univariate analysis of the first principal component of the traits (UV-PCA), and meta-analysis of univariate results (UV-MA). In UV-PCA, the first principal component of a standard principal component analysis is extracted and used in a univariate analysis. In UV-MA, p-values obtained in standard UV GWAS analyses are combined in a meta-analysis approach. Our goal was to provide researchers with insights that will guide the application of the methods to real data.

## Methods

### Data simulation

We simulated genotype and phenotype data for 1,000 individuals. Simulations were performed in R.

Genotype data were simulated for one bi-allelic quantitative trait locus (QTL) with minor allele frequency *q* and major allele frequency *p*. Genotypes were generated by sampling two alleles independently from a binomial distribution as 0 or 1, using two trials and a probability of success of each trial equal to *q*. The genotype is the sum of the two alleles, which can be 0, 1 or 2. Because alleles were sampled independently, genotypes were in Hardy Weinberg equilibrium.

Phenotype simulation was based on work by Saint-Pierre and colleagues [14]. For each individual, three quantitative traits Y_j_ (j = 1, 2, 3) were simulated. The trait-specific QTL heritabilities (the relative variance of Y_j_ explained by the QTL, h^2^_j_), MAF of the QTL *q*, and residual correlation between the traits excluding the QTL effect (rE_jj_) were controlled.

First, the effect of the QTL on the individual traits, a_j_, was determined from h^2^_j_ and *q* using the following formula [14]: 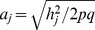


Secondly, the traits were constructed by adding up the trait-specific effect of the QTL and a residual component. Here, the trait-specific effect of the QTL was assumed to be additive and obtained by multiplying a_j_ with the number of effect (minor) alleles. The residual component 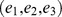 was simulated from a multivariate normal distribution with mean zero and with variance-covariance structure: 
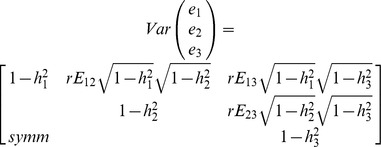


where 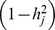 is the trait-specific proportion of the variance not explained by h^2^_j_ and rE_jj_ is the residual correlation between the traits excluding the QTL effect. The correlated residuals were generated using the function mvrnorm from the R package MASS. Third, all traits were centered and scaled to have zero mean and unit variance.

### Application of methods

#### MV-PLINK

The command used for association testing with MV-PLINK [7], [23] (https://genepi.qimr.edu.au/staff/manuelF/multivariate/main.html) was: *plink.multivariate —noweb —file geno —mqfam —mult-pheno pheno.phen —out output*. We applied an additive model. MV-PLINK produces an F-statistic and a p-value per genetic variant analyzed. This p-value of multivariate association was extracted from the output. Note that the canonical correlation analysis (CCA) applied by MV-PLINK is similar to multivariate analysis of variance (MANOVA) as CCA is applied to a single genetic variant at a time.

#### MV-SNPTEST

The command used to perform additive association testing with MV-SNPTEST [20] (https://mathgen.stats.ox.ac.uk/genetics_software/snptest/snptest.html#multiple_phenotype_tests) was: *snptest -data geno.gen pheno.sample -o output -bayesian 1 -method expected -mpheno T1 T2 T3 -prior_qt_mean_b 0 -prior_qt_V_b 0.02 -prior_mqt_c 4 -prior_mqt_Q 6*.

An inverse Wishart prior [IW(6,4)] was set on the error covariance matrix ∑ and a matrix normal prior [N(0.02,∑)] on the vector of parameters, according to recommendations of the authors. Method ‘expected’ was applied, which results in the use of expected genotype counts (∼dosages) in the analyses. The output file contains a log_10_ Bayes Factor (BF) per genetic variant, which was extracted for the purpose of this study.

#### MultiPhen

MultiPhen is an R package available from CRAN (http://cran.r-project.org/web/packages/MultiPhen/MultiPhen.pdf) for the R software (http://www.r-project.org/) [12]. The test for association is a likelihood ratio test (LRT) for model fit, testing whether all regression coefficients in the model are jointly significantly different from zero. We analyzed the simulation data using the mPhen function, specifying the genotype data, phenotype data and JointModel = TRUE. This results in a p-value per trait and a p-value for the LRT. The latter was extracted from the output.

#### MV-BIMBAM

The BIMBAM software [22] can be run in two different ways: 1) using option –mph 1, which tests for association between the multivariate traits, all partitioned in the group of directly affected traits, and genotype; and 2) using option –mph2, which considers all the different possible partitions of traits into the different categories of traits (directly affected, indirectly affected, unaffected). We applied option –mph2 under the additive model using the following command: *bimbam -g geno.txt -p pheno.txt -o output -f 3 -mph2 -A 0.1 -A 0.2*.

According to recommendations of the authors, the prior for the genetic effect A was set at 0.1 and 0.2. The association results are summarized by a log_10_ BF that evaluates presence of any association between the QTL and the traits averaging over all possible partitions of the traits into the different groups. This value was extracted from the output.

#### PCHAT

In PCHAT [4] (http://www.wpic.pitt.edu/wpiccompgen/PCHAT/PCHAT.htm) the sample is split in a training set, which is used to construct the optimal linear combination of traits from a heritability point of view, and a test set, which is used for association testing between genotype and the optimal linear combination of traits. In this way, use of the same data for both estimation of the optimal linear combination of traits and association testing is avoided. In addition, so called ‘bagging’ is performed, in which bootstrap samples are drawn from the training sample and the optimal linear combination of traits is averaged across bootstrap samples. The null distribution of the test statistic is obtained in the same way, using permutation of the data. We applied the additive model and set input parameters to values recommended by the authors: 50 subsets and bagging subsets for the determination of the distribution of the PCHAT test statistic under the null hypothesis; 200 and 50 subsets and bagging subsets, respectively, for testing the association of a genetic variant with the trait; 150 individuals for the subsets; and 1000 simulations for determination of the distribution of the test statistic under the null hypothesis. The option “both” was used for the analysis, resulting in a permutation experiment to determine the null distribution of the test statistic, after which the association test is performed using the standard deviation and degrees of freedom obtained in the permutation test. PCHAT produces 11 output files. In one of them, the association result is expressed as a p-value, which was extracted for this study.

#### TATES

TATES [15] (http://ctglab.nl/software) requires a correlation matrix of the traits and univariate association results as input. Full, symmetrical correlation matrices were generated using the corr option in R. UV analyses for the traits were performed by fitting linear models using the lm function in R. TATES was run in R using the freely available script specifying three traits and one genetic variant. The output contains the TATES trait-based p-value corrected for the correlations between the traits, which was extracted for this study.

#### Univariate (UV) analysis, meta-analysis of univariate results (UV-MA) and UV-PCA

UV analyses were performed as described under ‘TATES’. Resulting p-values were extracted for the purpose of this study. UV-MA was performed with METAL [24] (http://www.sph.umich.edu/csg/abecasis/Metal/), using univariate results per trait as input files and the analysis scheme ‘scheme samplesize’, which uses p-value and direction of effect as input for the MA and weighs according to sample size. PCA was performed in R using the princomp command. UV-PCA was executed using the first principal component (PC) in a univariate analysis as described above.

### Empirical significance thresholds (H0 simulations) and power

For each simulation scenario (see below) 1,000 datasets were simulated. Empirical significance thresholds for all methods were derived based on permutation of the traits generated in these simulation datasets, resulting in the null distribution of the test statistic. We generated 10 permuted datasets per simulation dataset, resulting in a total of 10,000 permuted replicates per scenario. These replicates were analyzed with the multivariate methods, resulting in a p-value or log_10_ BF for each replicate per method per scenario. Significance thresholds were set in such a way that 5% of the 10,000 replicates per method yielded a significant result (5% false-positive rate). This was done by sorting the 10,000 association measures in ascending (p-values) or descending order (Bayes Factors) and defining the empirical significance threshold as the mean of the 500^th^ and 501^st^ association measure.

UV analysis results in one p-value per trait. We adjusted the significance thresholds for three UV association tests, ensuring that alpha was fixed at 5% for all traits combined. For each null model, we first determined which trait was most strongly associated with the QTL per replicate, *i.e*. which trait resulted in the smallest p-value. These 10,000 p-values were sorted in ascending order and the mean of the 500^th^ and 501^st^ p-value was set as the threshold.

The power is defined as the percentage of 1,000 replicates for which the extracted p-value was smaller or log_10_ BF was larger than the empirical significance thresholds, ensuring an equal type I error rate of 5% for all methods.

### Simulation scenarios

Simulations were focused on three main scenarios in which one, two or three out of the three traits were associated with the QTL. Within these main scenarios, data sets were generated for a given combination of parameter values (rE_jj_, h^2^_j_, rG and *q*) as shown in Table 1. This resulted in a total of 30 simulation scenarios.

**Table 1:** Simulation scenarios.

# traits associated with QTL	Heritability (h^2^_j_)	Effect size (a_j_)	rG	rE	MAF (*q*)
1	h^2^_1_ = 0.1%, h^2^_2_ = h^2^_3_ = 0	a_1_>0, a_2_ = a_3_ = 0	0	3×0/3×0.3/3×0.7	0.01/0.4
2	h^2^_1_ = h^2^_2_ = 0.1%, h^2^_3_ = 0	a_1_ = a_2_, a_3_ = 0	+	3×0/3×0.3/3×0.7	0.01/0.4
	h^2^_1_ = h^2^_2_ = 0.1%, h^2^_3_ = 0	−a_1_ = a_2_, a_3_ = 0	−	3×0/3×0.3/3×0.7	0.01/0.4
3	h^2^_1_ = h^2^_2_ = h^2^_3_ = 0.1%	a_1_ = a_2_ = a_3_	+	3×0/3×0.3/3×0.7	0.01/0.4
	h^2^_1_ = h^2^_2_ = h^2^_3_ = 0.1%	−a_1_ = a_2_ = a_3_	−	3×0/3×0.3/3×0.7	0.01/0.4

MAF indicates minor allele frequency; j, trait; QTL, quantitative trait locus; rE, residual correlation; rG, genetic correlation.

We simulated positive residual correlations between the traits and studied scenarios with a relatively high and low residual correlation (rE_jj_ = 0.7 and rE_jj_ = 0.3, respectively). The QTL was fixed to explain 0.1% of the trait variances. By varying the sign of a_1_, we created a QTL induced correlation (rG) between trait 1 and traits 2 and 3 which was either positive or negative, enabling us to study the influence of a negative genetic correlation.

Note that due to the fixed trait-specific QTL heritabilities, the resulting QTL effects on the individual traits are larger for smaller q and vice versa. This fits with the scenario one would expect in real data [25].

## Results

### Empirical significance thresholds

Table S1 shows the empirical significance thresholds for all methods for every simulation scenario. Thresholds were around 5% for MV-PLINK, MultiPhen, TATES and UV-PCA. Significance thresholds for PCHAT were slightly increased to approximately 6%, indicating slight deflation of type I error rate under the null. MV-SNPTEST and MV-BIMBAM showed log_10_ BF significance thresholds between -0.05 and 0.44. Significance thresholds for UV-MA were highly dependent on the residual correlation between the traits: around 5% for scenarios with uncorrelated traits and 0.2-0.3% for scenarios with high residual correlation, thus indicating high inflation of type I error rate under the null for the latter scenarios. Thresholds for UV analysis were around 5%/3 = 1.7% for scenarios with no residual correlation and slightly increased with increasing residual correlation.

### Power comparison

#### One out of three traits associated with the QTL (Figure 2A)

All MV methods resulted in higher power than UV analysis. Power of MV-PLINK, MV-SNPTEST, MultiPhen and MV-BIMBAM was similar: between 10% and 15% for scenarios with a residual correlation of 0 or 0.3 and 20-25% for scenarios with rE = 0.7. PCHAT outperformed MV-PLINK, MV-SNPTEST, MultiPhen and MV-BIMBAM for scenarios with rE = 0 and 0.3, but not for scenarios with rE = 0.7, although power of PCHAT increased for rE = 0.7 as well. TATES showed a power between 11-14% for all scenarios and performed slightly better than UV-PCA and UV-MA, which showed a similar performance as UV analysis of trait 1, the trait associated with the QTL.

#### Two out of three traits associated with the QTL (Figure 2B)

MV-PLINK, MV-SNPTEST, MultiPhen and MV-BIMBAM showed the best and similar performance, with higher power with increasing residual correlation. This was most noticeable when the correlation induced by the QTL was negative.

PCHAT and TATES showed a robust performance relatively independent of the residual correlation, but their power never exceeded that of MV-PLINK, MV-SNPTEST, MultiPhen or MV-BIMBAM.

UV-PCA and UV-MA showed the same performance for scenarios in which there was a residual correlation between the traits. For scenarios with no residual correlation, UV-PCA performed better under negative genetic correlation and UV-MA under positive genetic correlation. They were outperformed by UV analysis of trait 1 and 2 only in case of a negative genetic correlation.

#### Three out of three traits associated with the QTL (Figure 2C)

Again, MV-PLINK, MV-SNPTEST, MultiPhen and MV-BIMBAM performed similar and best under simulation scenarios with a negative genetic correlation; in this case, their power increased with increasing residual correlation. However, for scenarios with a positive genetic correlation, power of MV-PLINK, MV-SNPTEST, MultiPhen and MV-BIMBAM increased with decreasing residual correlation.

PCHAT and TATES showed a comparable performance over all simulation scenarios. Also here, power slightly increased with decreasing residual correlation in case of a positive genetic correlation, and slightly increased with increasing residual correlation for a negative genetic correlation.

Similar to simulation scenarios with two out of three traits associated to the QTL, UV-PCA and UV-MA showed comparable power for all scenarios with rE>0. Again, for scenarios with rE = 0, UV-PCA performed better in case of a negative genetic correlation and UV-MA performed better in case of a positive genetic correlation. UV-MA outperformed all methods when rE was 0 and the genetic correlation was positive; for rE = 0.3 and a positive genetic correlation, UV-PCA and UV-MA performed best of all methods. Power of UV-MA was the same as that of UV analysis for all scenarios with a negative genetic correlation.

**Figure 2:**
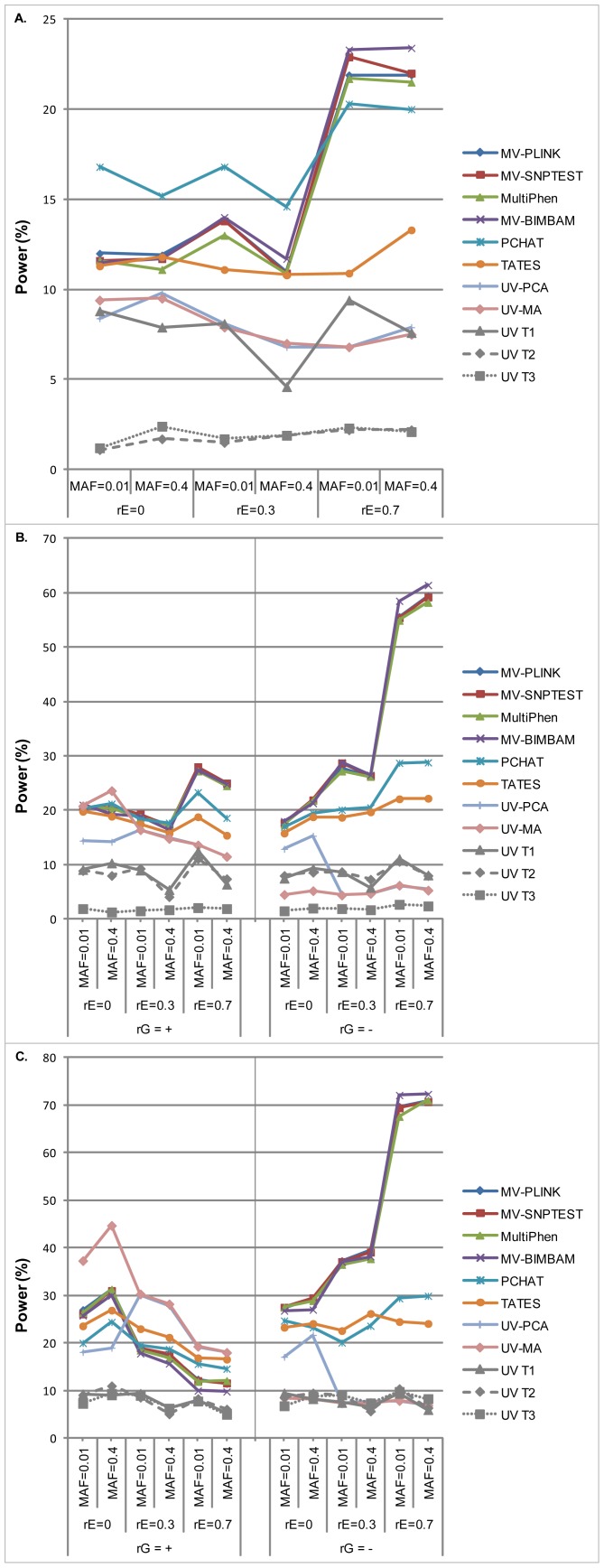
Power of the methods for scenarios with one of three traits associated with the QTL (A), two of three traits associated with the QTL (B) and with all three traits associated with the QTL (C). The explained variance of the QTL was fixed at 0.1%. For clarity reasons, we have not provided errors bars. Confidence ranges for the power estimates are all between 1 and 5%; exact values are provided in Tables S3–S5. MAF, minor allele frequency; MV, multivariate; PCHAT, Principal Component of Heritability Association Test; QTL, quantitative trait locus; rE, residual correlation; rG, genetic correlation induced by the QTL; TATES, Trait-based Association Test that uses Extended Simes procedure; UV-MA, meta-analysis of univariate results; UV-PCA, univariate analysis of first principal component; UV T1, univariate analysis of trait 1; UV T2, univariate analysis of trait 2; UV T3, univariate analysis of trait 3.

Results for simulation scenarios with different heritabilities for the three traits (h^2^_1_ = 0.001, h^2^_2_ = 0.002, h^2^_3_ = 0.0005) are comparable to the results presented in Figure 2C (Table S2).

Results for MAF = 0.25, 0.10, and 0.05 are shown in Tables S3-S5. As expected, power was similar for all MAF scenarios. Low MAF was not a problem for the methods, except for MultiPhen which experienced convergence problems of the underlying R function ‘polr’ when MAF was equal to or lower than 5%.

### Run time

Run time was measured on a Linux cluster using one core on a node equipped with 24 GB RAM and two Intel Xeon L5520 processors running on 2.26 GHz. Time for performing association analyses for 1000 subjects and 1000 replicates (similar to 1000 genetic variants in our study) was recorded. For TATES and UV-MA, run time also included the time used for UV association analyses of the three traits. MV-BIMBAM was the fastest method using 9 seconds, while PCHAT needed 437 minutes and 15 seconds. Run times for MV-PLINK, MV-SNPTEST, MultiPhen, TATES, UV-PCA and UV-MA were 23 seconds, 1 minute and 10 seconds, 3 minutes and 18 seconds, 23 seconds, 23 seconds and 19 seconds, respectively.

## Discussion

In this study, we used simulated data to compare the performance of six multivariate genome-wide association methods (MV-PLINK, MV-SNPTEST, MultiPhen, MV-BIMBAM, PCHAT and TATES) and standard univariate analysis, univariate PCA, and meta-analysis of univariate analyses. Our results showed that there is not a single method that performed best under all simulation scenarios. However, all six multivariate methods resulted in a higher power than UV analysis, even when only one of the traits was associated with the QTL. UV-MA only outperformed all methods when all traits were associated with the QTL and the genetic correlation was positive.

Use of multivariate GWAS can be recommended even when genetic correlations between traits are expected to be weak. Indeed, even when only one of the traits was associated with the QTL and thus in the absence of genetic correlation and pleiotropy, MV analyses resulted in higher power than UV analyses. This was described before by Liu *et al*. for bivariate analyses and is due to the differences in the penalty for multiple testing [9]. Note that this penalty is commonly not applied in multiple UV analyses of real data.

The influence of the strength of residual correlation, *i.e*. the relative amount of shared genetics, and sign of genetic correlation, *i.e*. difference in sign of QTL effect, on power varied across the different methods. For MV-PLINK, MV-SNPTEST, MultiPhen and MV-BIMBAM, higher power was observed with increasing residual correlation in case of a single QTL trait and when two or all three traits were associated with the QTL with a negative genetic correlation. The latter is due to the resulting opposite sign of genetic and residual correlation. Indeed, we observed a similar increase in power when simulating a positive genetic correlation and negative residual correlation (data not shown). This effect has been described before for these and other methods [7], [9], [12], [14] and was demonstrated analytically by Evans for bivariate linkage analysis [26]. In contrast, when residual and genetic correlation were in the same direction, power of these four methods decreased with increasing residual correlation between the traits, which also corroborates previous findings [7], [12]. PCHAT and TATES were relatively independent of the underlying (genetic) correlations of the traits. For PCHAT, this can be explained by the fact that it constructs the optimal linear combination of traits from an heritability point of view, thereby essentially removing the influence of residual correlation on power [4]. For TATES, it was described that the power was not influenced by opposite effects of the QTL on the traits, because of its reliance on p-value information [15]. UV-MA did however severely suffer from a negative genetic correlation between the traits; indeed, in this scenario it performed equal or worse than standard UV analysis. These findings are not unexpected; a negative genetic correlation between the traits is disastrous for the power of a MA, because the direction of effect is taken into account. An alternative meta-analysis approach is Fisher's method [27]. As it combines univariate p-values into one test statistic, similar to TATES, it does not suffer from a differential sign of effect. For scenarios with a negative genetic correlation, Fisher's method performed better than UV-MA and also better than TATES, except for scenarios with a residual correlation of 0.7: here it was outperformed by TATES but not by UV-MA (data not shown). The reduced performance for an opposite QTL effect was observed for UV-PCA as well, but not for scenarios with no residual trait correlation. In these scenarios, the first PC reflects the negative genetic correlation between the traits. Power is thus increased compared to UV since there is no need to multiple testing penalty.

We would like to emphasize that all methods were compared based on empirically derived significance levels, adjusting each method to an exact 5% type I error rate. Null simulations illustrated that for MV-PLINK, MV-SNPTEST, MultiPhen, MV-BIMBAM, TATES and UV-PCA these empirical significance levels were all close to the nominal level of 0.05 for p-values or between 0.01-1 for log_10_ BF [28]. PCHAT was slightly conservative based on our observations. Thresholds for UV analysis were around 1.7% (*i.e*. 5 divided by 3) in case of uncorrelated traits, which is in line with a Bonferroni correction for three independent tests. As expected, for correlated traits the adjusted threshold was less stringent and somewhere between 1.7 and 5%. We found that significance thresholds for UV-MA were highly dependent on trait correlations with increased stringency with increase in correlation. Trait correlations result in longer tails for the test statistic distribution, and therefore a more stringent threshold must be applied to keep the type I error at 5%. Thus, use of the UV-MA can potentially lead to a high number of false-positive findings if traits are highly correlated and the significance threshold is not appropriately adjusted.

Some of the methods included in our study have been compared to one another before. MV-PLINK and MultiPhen were compared by O'Reilly *et al*. and van der Sluis *et al*., which showed that both methods result in the same power when restricting the analysis to normally distributed traits [12], [15], corroborating our findings. TATES was compared to MANOVA (which is similar to MV-PLINK) and shown to be only outperformed by MANOVA in the particular condition that the genetic variant affects only one of multiple strongly correlated traits [15]. In contrast, we observed that MV-PLINK outperformed TATES in almost all scenarios.

In addition to power (and type I errors), there are other characteristics that are important to take into account when deciding upon the appropriate multivariate GWAS analysis. MV-PLINK output results contain trait loadings, which indicate how much each trait contributed to the multivariate association result [7]. MV-BIMBAM outputs marginal posterior probabilities for each trait being unaffected, directly affected or indirectly affected by the QTL, conditional on an overall association with at least one trait [22]. PCHAT gives the weights for each of the traits included in the analysis which were used to construct the optimal linear combination of the traits to detect an association with the QTL [4]. MultiPhen output contains the betas and p-values for the association of each trait with the QTL based on the joint model including all traits [12]. This additional information, which is not provided by MV-SNPTEST and TATES, can be used to obtain insight into underlying biology and facilitates the discrimination between independent and pleiotropic QTL effects. Furthermore, MV-PLINK, MultiPhen, TATES and UV-MA allow analysis of a combination of quantitative and binary (case-control) traits [7], [12], [15]. MV-BIMBAM and UV-MA can be applied to summary data, without access to raw phenotype and genotype data [22]. Also, MV-SNPTEST, MultiPhen, TATES, UV-PCA and UV-MA are able to handle genotype probabilities as obtained by imputation while the other methods are not [12], [15], [20]. Finally, our study showed large differences in run time between the methods.

Our simulations are not exhaustive. Data were simulated for three traits according to an additive model, and analyzed accordingly. We did not simulate and analyze other, non-additive genetic models and/or (higher-order) interactions, nor did we study scenarios with more than three traits. In addition, priors for MV-SNPTEST and MV-BIMBAM and input parameters (*e.g*. number of (bagging) subsets) for PCHAT were not varied. Also, we did not study the effect of missing data. This was explored by Klei *et al*. for PCHAT who concluded that dropping individuals with missing data had a substantial diminishing effect on power of the test [4]. In addition, Van der Sluis *et al*. [15] reported that 10% missingness completely at random hardly affected the power to detect QTLs when the QTL affected all traits, but that it resulted in a higher power drop for MANOVA compared to TATES when the QTL was only associated to one of the traits. Finally, we did not simulate trait outliers in our data. O'Reilly showed that this could result in substantial inflation of the statistics for CCA for low frequency variants [12]. However, in our opinion outliers should be handled appropriately prior to association analyses.

Taken together, our study showed substantial differences in power between the methods, dependent on the simulation scenario. For some of the simulation scenarios, a large increase in power of multivariate compared to univariate analyses was observed, which suggests that the multivariate methods might be able to identify genetic variants that are currently not identifiable by standard univariate analysis. Overall, MV-PLINK, MV-SNPTEST, MultiPhen and MV-BIMBAM performed best for the majority of the tested scenarios, with a remarkable increase in power for scenarios with an opposite sign of genetic and residual correlation. As a consequence, results of these methods will be biased towards QTLs that cause a genetic correlation that is opposite in sign to the residual correlation. PCHAT and TATES showed a robust performance over all simulation scenarios and are therefore recommended to use if one aims to obtain a reflection of the underlying genetic architecture of the traits.

## Supporting Information

Table S1 **Empirical significance thresholds of the methods for every simulation scenario.**
(XLSX)

Table S2 **Power of the multivariate GWA methods for scenarios with all three traits associated with the QTL with an explained variance fixed at 0.1% for trait 1, 0.2% for trait 2 and 0.05% for trait 3.**
(XLSX)

Table S3 **Power of the multivariate GWA methods for scenarios with one out of three traits associated with the QTL with an explained variance fixed at 0.1%.**
(XLSX)

Table S4 **Power of the multivariate GWA methods for scenarios with two out of three traits associated with the QTL with an explained variance fixed at 0.1%.**
(XLSX)

Table S5 **Power of the multivariate GWA methods for scenarios with all three traits associated with the QTL with an explained variance fixed at 0.1%.**
(XLSX)
